# Prevalence and Associated Factors of Depressive Symptoms Among Patients With Chronic Low Back Pain: A Cross-Sectional Study

**DOI:** 10.3389/fpsyt.2021.820782

**Published:** 2022-01-13

**Authors:** Yueming Hu, Zechuan Yang, Yong Li, Yong Xu, Mengge Tian, Nan Jiang, Ningfeng Guo

**Affiliations:** ^1^Department of Orthopedics, Hwa Mei Hospital, University of Chinese Academy of Sciences, Ningbo, China; ^2^Ningbo Institute of Life and Health Industry, University of Chinese Academy of Sciences, Ningbo, China; ^3^Department of Orthopedics, Tongji Hospital, Tongji Medical College, Huazhong University of Science and Technology, Wuhan, China; ^4^Department of Social Medicine and Health Management, School of Public Health, Tongji Medical College, Huazhong University of Science and Technology, Wuhan, China

**Keywords:** depressive symptoms, chronic low back pain, prevalence, risk factors, patients

## Abstract

**Background:** Few studies have focused on depressive symptoms among patients with chronic low back pain in China. The aim of this cross-sectional study was to assess the prevalence and associated factors of depressive symptoms in patients with chronic low back pain.

**Methods:** From May to August 2021, 1,172 patients with chronic low back pain were recruited in China. Depressive symptoms were assessed through the Patient Health Questionnaire. Associations of demographic characteristics, clinical characters and social-psychological factors with depressive symptoms were investigated among patients with chronic low back pain.

**Results:** The prevalence of depressive symptoms was 25.00%. Logistic regression analysis found that duration of pain in 1-5 years (1-3 years: OR = 2.91, 95%CI: 1.65-5.14, 3-5 years: OR = 3.09, 95%CI: 1.55-6.15) and more severe pain (OR = 1.13, 95%CI: 1.10-1.17) were associated with higher risks of depressive symptoms. Better family function (good family function: OR = 0.25, 95%CI: 0.15-0.41, moderate family dysfunction: OR = 0.47, 95%CI: 0.29-0.77) and higher pain self-efficacy (OR = 0.94, 95%CI: 0.93-0.95) were associated with lower risks of depressive symptoms.

**Conclusion:** Patients with chronic low back pain have a high prevalence of depressive symptoms in China. Duration of pain, pain severity, family function and pain self-efficacy were predictors of depressive symptoms among chronic low back pain patients in China. Early identification of the associated factors may be helpful for the timely management of depressive symptoms.

## Introduction

Low back pain is a worldwide public health issue (1). And of which, chronic low back pain (CLBP), as a chronic health problem, has attracted much attention due to its features of persistent suffering, high disability rate and heavy medical burden (2). Studies have shown that the prevalence of CLBP has been increasing in recent years, with some countries reaching over 20%, such as Brazil (25%) and Norway (23.6%) (2–4). CLBP not only causes long-term suffering for patients, impairs their physical function and even results in disability, but also increases personal and social medical costs (5–7). Moreover, with the prolongation of pain and the progress of the disease, the risk of psychological problems in patients with CLBP also increased significantly (6).

Depressive symptoms are the most common mental health problem in patients with CLBP (8). And the prevalence of depressive symptoms in patients with CLBP is higher than that of the general population. A previous large-scale study using South Korea national representative population data showed that the prevalence of depressive symptoms in patients with CLBP was as high as 20.3%, which is much higher than 4.5% of the general population (9). A longitudinal study in Spain also showed that patients with chronic pain (such as CLBP) were 1.4 times more likely to have depressive symptoms than patients without chronic pain (8). CLBP patients with depressive symptoms will not only increase the risk of disability and decrease the quality of life, but also lead to an increase in medical costs (10–12). A previous study revealed that CLBP patients with severe depressive symptoms have a higher disability index, and depressive symptoms are the most powerful explanation for their increased disability index (11). A systematic review also found that the direct medical costs of CLBP patients with psychiatric disorders have increased significantly (12).Therefore, it is necessary to explore the prevalence of depressive symptoms and related factors in CLBP patients, thereby developing effective intervention strategies.

China has a large group of patients with CLBP due to its large population base. According to the literature, 67.3 million people in China suffered from CLBP in 2016, causing a heavy burden of disease (13). The results of the Global Burden of Disease study also showed that CLBP was the leading cause of years lived with disability in China, and it is of great significance for patients and society to have a good management of CLBP (14). However, there has been little research on the prevalence and associated factors of depressive symptoms in CLBP patients in China. Therefore, this study aimed to investigate the prevalence of depressive symptoms in CLBP patients attending hospitals and explore its related factors to provide relevant evidence for promoting the mental health of CLBP patients.

## Materials and Methods

### Ethics Statement

The study was approved by the Ethics Committee of Ningbo No.2 Hospital, Zhejiang, China. All participants were voluntary and provided written informed consent before being involved in the study. All data of participants were kept anonymous and confidential.

### Participants and Sampling

This study adopted the convenience sampling method to select a general hospital in Ningbo, Zhejiang province and Wuhan, Hubei province in China, respectively, as the study sites. Patients with CLBP admitted to Tongji Hospital in Wuhan and the Ningbo No.2 Hospital in Ningbo during May to August 2021 were invited to participate in this survey. The inclusion criteria included: (1) aged ≥ 18 years, (2) duration of low back pain ≥ 3 months, (3) a native Chinese speaker, (4) volunteer to participate in this survey. Participants were excluded if they: (1) had a trauma or surgery on the low back within the past year, (2) had a pregnancy within the past year, (3) diagnosed severe psychiatric diseases.

We calculated the minimum sample size by a single population proportion formula: *n* = $z_{\alpha /2}^{2}p\left(1-p\right)/d^{2}$, where *n* is the sample size, p is the proportion of depressive symptoms among CLBP patients, z is the normal deviation, and d is the margin of error. In order to control type I error, we took α as 0.05; therefore, z was equal to 1.96. Based on previous research on the depressive symptoms among CLBP patients, p was assumed to be 20.3% (9). Besides, we took d as 0.15p. The calculated minimum sample size was 670. In this study, allowing a non-response rate of 20% and the final sample size was 838.

### Measures

The Patient health questionnaire (PHQ-9) was used to assess depressive symptoms, which consisted of 9 items (15). Each item is evaluated by the frequency of the symptom over the last 2 weeks: “Not at all” scored 0, “Several days” scored 1, “More than half the days” scored 2, and “Nearly every day” scored 3. The total score ranges from 0 to 27, with a cut-off point of 10 differentiating depressive symptom and no depressive symptom. The PHQ-9 has been validated in Chinese patients with good reliability and validity in previous studies (16, 17). In this study, Cronbach's alpha coefficient of this scale was 0.94.

To control for the influence of potential confounding factors on depressive symptoms, we collected information on demographic characteristics, clinical characters (pain severity and duration of pain), family functioning, and pain self-efficacy. The demographic characteristics included sex, age, work status, marital status, educational level, monthly personal income, and medical insurance.

The four-item Chinese version Brief Pain Inventory subscale on pain severity (BPI-PS) was used to assess pain intensity (18). The scale has been widely used in patients with low back pain (19). The four items of BPI-PS included pain severity at “its worst” over the past 24 h, “its least” over the past 24 h, “on average” over the past 24 h, and “right now”. Patients were asked to rate the extent to pain from 0 (no pain) to 10 (pain as bad as you can imagine) at above four moments. The total score of BPI-PS ranges from 0 to 40, with higher scores indicating more severe pain. In this study, the BPI-PS had a good internal consistency (Cronbach's alpha = 0.92).

The Chinese version of the Family Adaptation, Partnership, Growth, Affection, Resolve (APGAR) index was used to evaluate participants' satisfaction with current family function, which consisted of five items, namely, adaptability, partnership, growth, affection, and resolve (20, 21). Each item was scored 0, 1, and 2 according to its response “Hardly Ever,” “Some of the time,” and “Almost Always.” The total score ranges from 0 to 10 and has been divided into three levels: 0-3 indicates severe family dysfunction, 4-6 indicates moderate family dysfunction, and 7-10 indicates good family function (22, 23). Previous studies have proven to excellent validity and reliability of the scale in Chinese families (22, 23). In this study, Cronbach's alpha coefficient of this scale was 0.88.

Pain self-efficacy was measured through the Chinese version of the Pain Self-efficacy Questionnaire (PSEQ), which has shown excellent validity and reliability in patients with CLBP (24, 25). PSEQ is a psychometric scale, which reflects an individual's confidence in completing daily tasks despite experiencing pain. The scale consists of 10 items rated on a seven-point Likert scale from 0 (lowest confidence) to 6 (highest confidence). Hence, the total score for PSEQ ranges from 0 to 60, where high scores reflect higher pain self-efficacy. In this study, the scale demonstrated good internal consistency (Cronbach's alpha = 0.98).

### Statistical Analysis

The normality of continuous variables was test by the Shapiro-Wilk method. Due to the distribution of all continuous variables did not satisfy the normality (*P* < 0.05), continuous variables were showed by medians and quartiles. Categorical variables were showed by frequencies and percentages. We used Wilcoxon rank-sum test for continuous variables, and Pearson's chi-squared test for categorical variables to compare the demographic characteristics, pain-related variables, and family function between patients with or without depressive symptoms. The binary logistic regression was conducted to assess the association between outcomes and different explanatory variables. We also used stepped modeling approach to compare the fit of three sets of variables. Model 1 only contained individual characteristics; Model 2 contained both individual and pain-related factors; Model 3 was a full model which also included family function. Parameters including −2log likelihood, Akaike's information criterion (AIC) and Schwarz criterion (SC) were used to compare these multilevel models. The −2log likelihood, AIC and SC of Model 1, Model 2, and Model 3 became smaller, indicating that the fit of the models improved (Supplementary Table S1). The Hosmer-Lemeshow goodness-of-fit test was used to examine model fitness, and its *P*-value was >0.05 (*P* = 0.9257). The strength of association was interpreted using odds ratio (OR) and 95% confidence interval (CI). All *P*-values were two-tailed and significance level was set at 0.05. The statistical analysis was conducted using SAS 9.4 software.

## Results

A total of 1,238 patients with CLBP who met the inclusion criteria were included in this study, and 66 patients who did not complete the questionnaire were excluded. Ultimately, 1,172 patients were included in the analysis. The characteristics of participants and their association with depressive symptoms are presented in Table 1. The median age of the participants was 50.5 years, and the percentage of male was 50.00%. Most participants were married (82.08%), secondary school (29.69%), non-manual worker (38.57%), low-income group (58.19%), with medical insurance (89.16%), with good family function (54.86%), with the duration of pain <6 months (35.32%). The median score of the participants with respect to pain severity and pain self-efficacy was 12 and 41, respectively.

**Table 1:** Demographic and clinical characteristics of patients with chronic low back pain according to presence of depressive symptoms.

	**All patients**	**No depressive symptoms**	**Depressive symptoms**	***P-*value**
Age	50.5 (38-62)	50 (36-61)	52 (43-64)	0.0002^*^
Sex				0.0685
Male	586 (50.00%)	453 (51.54%)	133 (45.39%)	
Female	586 (50.00%)	426 (48.46%)	160 (54.61%)	
Marital status				0.0947
Unmarried/divorce/widowed	210 (17.92%)	148 (16.84%)	62 (21.16%)	
Married	962 (82.08%)	731 (83.16%)	231 (78.84%)	
Educational level				<0.0001^**^
Primary school or below	268 (22.87%)	194 (22.07%)	74 (25.26%)	
Secondary school	348 (29.69%)	233 (26.51%)	115 (39.25%)	
Senior high school	177 (15.10%)	135 (15.36%)	42 (14.33%)	
Junior college	172 (14.68%)	146 (16.61%)	26 (8.87%)	
University or above	207 (17.66%)	171 (19.45%)	36 (12.29%)	
Work status				0.1865
Unemployed	377 (32.17%)	272 (30.94%)	105 (35.84%)	
Manual worker	343 (29.27%)	256 (29.12%)	87 (29.69%)	
Non-manual worker	452 (38.57%)	351 (39.93%)	101 (34.47%)	
Monthly personal income				<0.0001^**^
< ¥5,000	682 (58.19%)	474 (53.92%)	208 (70.99%)	
≥¥5,000	490 (41.81%)	405 (46.08%)	85 (29.01%)	
Medical insurance				<0.0001^**^
Yes	1,045 (89.16%)	811 (92.26%)	234 (79.86%)	
No	127 (10.84%)	68 (7.74%)	59 (20.14%)	
Duration of pain				<0.0001^**^
<6 months	414 (35.32%)	280 (31.85%)	134 (45.73%)	
6 months to 1 years	288 (24.57%)	245 (27.87%)	43 (14.68%)	
1 to 3 years	263 (22.44%)	207 (23.55%)	56 (19.11%)	
3 to 5 years	105 (8.96%)	71 (8.08%)	34 (11.60%)	
>5 years	102 (8.70%)	76 (8.65%)	26 (8.87%)	
Pain severity	12 (9-19)	11 (8-16)	22 (14-25)	<0.0001^**^
Family function				<0.0001^**^
Good family function	643 (54.86%)	537 (61.09%)	106 (36.18%)	
Moderate family dysfunction	377 (32.17%)	273 (31.06%)	104 (35.49%)	
Severe family dysfunction	152 (12.97%)	69 (7.85%)	83 (28.33%)	
Pain self-efficacy	41 (27-51.5)	47 (36-53)	21 (5-38)	<0.0001^**^

* *P-value < 0.05,*

** *P-value < 0.001*.

In this study, the prevalence of depressive symptoms in participants was 25.00% (*n* = 292). The univariable analysis showed that age, educational level, monthly personal income, medical insurance, duration of pain, family function, the score of pain severity and pain self-efficacy were significantly different between patients with and without depressive symptom (Table 1).

Logistic regression analysis showed that duration of pain, pain severity, family function, pain self-efficacy was significantly associated with depressive symptoms. The results found that patients with pain duration of 1-3 years (OR = 2.91, 95% CI: 1.65-5.14) and 3-5 years (OR = 3.09, 95% CI: 1.55-6.15) were more likely to suffer from depressive symptoms than those with a duration of <6 months. Patients with CLBP who had higher scores of pain severity were at higher risk of developing depressive symptoms (OR = 1.13, 95% CI: 1.10-1.17), while higher score of pain self-efficacy was associated with a decreased likelihood of depressive symptoms (OR = 0.94, 95% CI: 0.93-0.95). Compared to those with severe family dysfunction, those with good family function (OR = 0.25, 95% CI: 0.15-0.41) and moderate family dysfunction (OR = 0.47, 95 % CI: 0.29-0.77) were less likely to have depressive symptoms (Table 2).

**Table 2:** Binary Logistic analysis of depressive symptoms related factors in patients with chronic low back pain.

	***OR* (95%*CI*)**	***P-*value**
Age	1.01 (0.99-1.03)	0.3800
Sex (Ref = “male”)		
Female	0.99 (0.69-1.43)	0.9633
Marital status (Ref = “Unmarried/divorce/widowed”)		
Married	0.77 (0.48-1.25)	0.2916
Educational level (Ref = “Primary school or below")		
Secondary school	1.25 (0.74-2.12)	0.4006
Senior high school	0.91 (0.46-1.79)	0.7900
Junior college	0.80 (0.34-1.87)	0.6016
University or above	1.16 (0.47-2.88)	0.7424
Work status (Ref = “Unemployed”)		
Manual worker	1.34 (0.82-2.21)	0.2429
Non-manual worker	1.24 (0.69-2.24)	0.4669
Monthly personal income (Ref = “ < ¥5,000”)		
≥¥5,000	0.67 (0.42-1.07)	0.0969
Medical insurance (Ref = “Yes”)		
No	1.46 (0.84-2.56)	0.1810
Duration of pain (Ref = “ <6 months”)		
6 months to 1 years	1.10 (0.62-1.96)	0.7465
1-3 years	2.91 (1.65-5.14)	0.0002^*^
3-5 years	3.09 (1.55-6.15)	0.0013^*^
>5 years	1.33 (0.67-2.63)	0.4152
Pain severity	1.13 (1.10-1.17)	<0.0001^**^
Family function (Ref = “severe family dysfunction”)		
Good family function	0.25 (0.15-0.41)	<0.0001^**^
Moderate family dysfunction	0.47 (0.29-0.77)	0.0028^*^
Pain self-efficacy	0.94 (0.93-0.95)	<0.0001^**^

* *P-value < 0.05,*

** *P-value < 0.001, Hosmer and Lemeshow goodness of fit test (P-value = 0.9257)*.

## Discussion

The results of this study showed that the prevalence of depressive symptoms in patients with CLBP in China was 25.00%, significantly higher than that in Chinese community-dwelling population (12.30%) (26). This finding was consistent with previous studies (9, 27). The high prevalence of depressive symptoms may be related to the pain of the disease and its negative effects on life (28). Compared with international studies of depressive symptoms in CLBP patients using the PHQ-9 to assess depressive symptoms, our result was higher than those in Korea (20.3%) (9) and Japan (16.47%) (28), which also suggested that health authorities should pay more attention to the problem of severe depressive symptoms in patients with CLBP in China.

Notably, there is no significant association between medical insurance and depressive symptoms in CLBP patients in this study. This result is contrary to studies in some countries in Southeast Asia (29, 30). This may be because China started to establish a basic medical insurance system covering all people in 2009. Statistics showed that the basic medical insurance coverage rate in China has been as high as 95% by 2020 (31). This is consistent with nearly 90% of the participants in this study with medical insurance.

Pain-related factors were significant related to depressive symptoms in CLBP patients. Our study found that CLBP patients who had pain duration of 1-5 years were at higher risk of developing depressive symptoms, which was similar to previous studies (32, 33). The reason may be that long-term low back pain has a negative impact on the patients' family, social and work activities, which can lead to more prone to depressive symptoms (33). However, in this study, the risk of depressive symptoms did not increase in CLBP patients with a duration of pain more than 5 years. One possible explanation is that as the duration of disease increases, patients may regard pain as more normative and adopt aggressive coping strategies, mitigating the negative effects of disease to physical and psychological health (34). Pain intensity was a risk factor for depressive symptoms in CLBP patients. The results of two previous studies in Japanese CLBP patients showed that more severe pain was associated with worse mental health conditions (28, 35). This suggests that while strengthening pain management of patients with CLBP, it is also important to focus on the psychological status of patients with pain duration of 1-5 years and higher pain severity, thereby timely identifying their psychological problems and providing psychological guidance services.

This study also found that family function was a significant protective factor for depressive symptoms (36–38). When CLBP patients are in a state of family dysfunction, they are more likely to cope with their illness negatively due to conflicting family roles, poor communication, and hampered problem solving, which makes them vulnerable to depressive symptoms (39). In contrast, when family function is good, CLBP patients will get more support from their family members and better cope with the disease and its related adverse emotions (39). In addition, CLBP patients with higher pain self-efficacy had a lower risk of depressive symptoms. Pain self-efficacy refers to the confidence or belief in one's ability to achieve behavioral goals in the context of pain (24). Patients with high levels of pain self-efficacy may make it easier for them to acknowledge the objective reality of the existence of CLBP, to actively manage and cope with the various adverse stresses associated with CLBP, with a correspondingly significantly lower risk of pain-related distress and psychological disorders (40). Therefore, the risk of depressive symptoms in patients with CLBP can be reduced by improving family function and pain self-efficacy.

To our best knowledge, this study is the first to investigate the prevalence of depressive symptoms in Chinese patients with CLBP and to explore the influencing factors in terms of demographic characteristics, clinical characters, individual psychological factors and social factors, which provides evidence for the prevention and treatment of depressive symptoms in patients with CLBP. However, there are some limitations of this study. First, this was a cross-sectional study, and it was difficult to clarify the causal relationship between depressive symptoms and related factors. Second, the convenience sample was adopted in this study, which limited representativeness and generalizability of the results. Third, this study was conducted in two provinces of China, and thus, the generalizability of our results to patients with CLBP in other regions of China may be limited. Further large-scale studies are needed on a national scale.

## Conclusion

The prevalence of depressive symptoms is high among Chinese patients with CLBP. In the clinic, CLBP patients with pain duration of 1-5 years should be paid more attention. Moreover, pain should be actively managed to reduce pain intensity in CLBP patients. In addition to the treatment of somatic pathological changes, the psychological well-being of CLBP patients should also be promoted by enhancing patients' family function and pain self-efficacy.

## Data Availability Statement

The original contributions presented in the study are included in the article/Supplementary Material. Further inquiries can be directed to the corresponding author.

## Ethics Statement

The study was approved by the Ethics Committee of Ningbo No.2 Hospital, Zhejiang, China. Participation was voluntary and all participants provided written informed consent before being involved in the study. All data of participants were kept anonymous and confidential.

## Author Contributions

YH, ZY, and NG were responsible for the conception, design, and writing of the manuscript. YL and YX were responsible for the acquisition of data and literature research. MT and NJ were responsible for the analysis and interpretation of data. All authors read and approved the final manuscript.

## Conflict of Interest

The authors declare that the research was conducted in the absence of any commercial or financial relationships that could be construed as a potential conflict of interest.

## Publisher's Note

All claims expressed in this article are solely those of the authors and do not necessarily represent those of their affiliated organizations, or those of the publisher, the editors and the reviewers. Any product that may be evaluated in this article, or claim that may be made by its manufacturer, is not guaranteed or endorsed by the publisher.

## Abbreviations

CLBP: chronic low back pain
PHQ-9: Patient health Questionnaire
BPI-PS: Brief Pain Inventory subscale on pain severity
APGAR, Adaptation, Partnership, Growth, Affection: Resolve
PSEQ: Pain Self-efficacy Questionnaire
OR: odds ratio
CI: confidence interval.

## References

[1] Hoy D Bain C Williams G March L Brooks P Blyth F. A systematic review of the global prevalence of low back pain. Arthritis Rheum. (2012) 64:2028-37. 10.1002/art.34347 22231424

[2] Alleva J Hudgins T Belous J Kristin Origenes A. Chronic low back pain. Dis Mon. (2016) 62:330-3. 10.1016/j.disamonth.2016.05.012 27344193

[3] Leopoldino AA Diz JB Martins VT Henschke N Pereira LS Dias RC. Prevalence of low back pain in older Brazilians: a systematic review with meta-analysis. Rev Bras Reumatol Engl Ed. (2016) 56:258-69. 10.1016/j.rbr.2016.01.004 27267645

[4] Heuch I Hagen K Heuch I Nygaard O Zwart JA. The impact of body mass index on the prevalence of low back pain. the HUNT study. Spine. (2010) 35:764-8. 10.1097/BRS.0b013e3181ba1531 20228714

[5] Last AR Hulbert K. Chronic low back pain: evaluation and management. Am Fam Physician. (2009) 79:1067-74. 10.1080/20786204.2010.10873969 19530637

[6] Kamper SJ Apeldoorn AT Chiarotto A Smeets RJ Ostelo RW Guzman J. Multidisciplinary biopsychosocial rehabilitation for chronic low back pain: cochrane systematic review and meta-analysis. BMJ. (2015) 350:h444. 10.1136/bmj.h444 25694111 PMC4353283

[7] Manchikanti L Singh V Falco FJ Benyamin RM Hirsch JA. Epidemiology of low back pain in adults. Neuromodulation. (2014) 17(Suppl 2):3-10. 10.1111/ner.12018 25395111

[8] Fernandez M Colodro-Conde L Hartvigsen J Ferreira ML Refshauge KM Pinheiro MB. Chronic low back pain and the risk of depression or anxiety symptoms. insights from a longitudinal twin study. Spine J. (2017) 17:905-12. 10.1016/j.spinee.2017.02.009 28267634

[9] Park SM Kim HJ Jang S Kim H Chang BS Lee CK. Depression is closely associated with chronic low back pain in patients over 50 years of age: a cross-sectional study using the Sixth Korea National Health and Nutrition Examination Survey (KNHANES VI-2). Spine. (2018) 43:1281-8. 10.1097/BRS.0000000000002595 29462063

[10] Keeley P Creed F Tomenson B Todd C Borglin G Dickens C. Psychosocial predictors of health-related quality of life and health service utilisation in people with chronic low back pain. Pain. (2008) 135:142-50. 10.1016/j.pain.2007.05.015 17611036

[11] Hung CI Liu CY Fu TS. Depression: an important factor associated with disability among patients with chronic low back pain. Int J Psychiatry Med. (2015) 49:187-98. 10.1177/0091217415573937 25930736

[12] Baumeister H Knecht A Hutter N. Direct and indirect costs in persons with chronic back pain and comorbid mental disorders–a systematic review. J Psychosom Res. (2012) 73:79-85. 10.1016/j.jpsychores.2012.05.008 22789408

[13] Wu A Dong W Liu S Cheung JPY Kwan KYH Zeng X. The prevalence and years lived with disability caused by low back pain in China, 1990 to 2016: findings from the global burden of disease study 2016. Pain. (2019) 160:237-45. 10.1097/j.pain.0000000000001396 30234697 PMC6319591

[14] Global Burden of Disease Study 2013 Collaborators. Global, regional, and national incidence, prevalence, and years lived with disability for 301 acute and chronic diseases and injuries in 188 countries, 1990-2013: a systematic analysis for the Global Burden of Disease Study 2013. Lancet. (2015) 386:743-800. 10.1016/S0140-6736(15)60692-4 26063472 PMC4561509

[15] Kroenke K Spitzer RL Williams JB. The PHQ-9: validity of a brief depression severity measure. J Gen Intern Med. (2001) 16:606-13. 10.1046/j.1525-1497.2001.016009606.x 11556941 PMC1495268

[16] Sun Y Fu Z Bo Q Mao Z Ma X Wang C. The reliability and validity of PHQ-9 in patients with major depressive disorder in psychiatric hospital. BMC Psychiatry. (2020) 20:474. 10.1186/s12888-020-02885-6 32993604 PMC7525967

[17] Feng Y Huang W Tian TF Wang G Hu C Chiu HF. The psychometric properties of the Quick Inventory of Depressive Symptomatology-Self-Report (QIDS-SR) and the Patient Health Questionnaire-9 (PHQ-9) in depressed inpatients in China. Psychiatry Res. (2016) 243:92-6. 10.1016/j.psychres.2016.06.021 27376668

[18] Wang XS Mendoza TR Gao SZ Cleeland CS. The Chinese version of the Brief Pain Inventory (BPI-C): its development and use in a study of cancer pain. Pain. (1996) 67:407-16. 10.1016/0304-3959(96)03147-8 8951936

[19] Chiarotto A Maxwell LJ Ostelo RW Boers M Tugwell P Terwee CB. Measurement properties of visual analogue scale, numeric rating scale, and pain severity subscale of the brief pain inventory in patients with low back pain: a systematic review. J Pain. (2019) 20:245-63. 10.1016/j.jpain.2018.07.009 30099210

[20] Good M-JD Smilkstein G Good BJ Shaffer T Arons T. The family APGAR index. a study of construct validity. J Fam Pract. (1979) 8:577-82.

[21] Chau TT Hsiao TM Huang CT Liu HW. [A preliminary study of family Apgar index in the Chinese]. Kaohsiung J Med Sci. (1991) 7:27-31. 1990150

[22] Huang Y Liu Y Wang Y Liu D. Family function fully mediates the relationship between social support and perinatal depression in rural Southwest China. BMC Psychiatry. (2021) 21:151. 10.1186/s12888-021-03155-9 33711987 PMC7953569

[23] Chen YM Yu TW Wang CC Huang KT Hsu LW Lin CC. Characteristics of psychosocial factors in liver transplantation candidates with alcoholic liver disease before transplantation: a retrospective study in a single center in Taiwan. Int J Environ Res Public Health. (2020) 17:8696. 10.3390/ijerph17228696 33238532 PMC7700481

[24] Nicholas MK. The pain self-efficacy questionnaire: taking pain into account. Eur J Pain. (2007) 11:153-63. 10.1016/j.ejpain.2005.12.008 16446108

[25] Xiao J Wu WX Teng HL Wang NN. Testing the reliablility and validity of Chinese version pain self-efficacy questionnaire in a population with chronic low back pain. J Nurs Sci. (2013) 28:32-4. 10.3870/hlxzz.2013.03.032

[26] Ma L Tang Z Zhang L Sun F Li Y Chan P. Prevalence of frailty and associated factors in the community-dwelling population of China. J Am Geriatr Soc. (2018) 66:559-64. 10.1111/jgs.15214 29168883

[27] Bener A Verjee M Dafeeah EE Falah O Al-Juhaishi T Schlogl J. Psychological factors. anxiety, depression, and somatization symptoms in low back pain patients. J Pain Res. (2013) 6:95-101. 10.2147/JPR.S40740 23403693 PMC3569050

[28] Montgomery W Vietri J Shi J Ogawa K Kariyasu S Alev L. The relationship between pain severity and patient-reported outcomes among patients with chronic low back pain in Japan. J Pain Res. (2016) 9:337-44. 10.2147/JPR.S102063 27330326 PMC4898257

[29] Ogbo FA Mathsyaraja S Koti RK Perz J Page A. The burden of depressive disorders in South Asia, 1990-2016: findings from the global burden of disease study. BMC Psychiatry. (2018) 18:333. 10.1186/s12888-018-1918-1 30326863 PMC6192293

[30] Misra S Wyatt LC Wong JA Huang CY Ali SH Trinh-Shevrin C. Determinants of depression risk among three asian american subgroups in New York City. Ethn Dis. (2020) 30:553–62. 10.18865/ed.30.4.553 32989355 PMC7518536

[31] China Daily. Moving the Agenda Forward. (2021). Available online at: https://www.chinadaily.com.cn/a/202107/16/WS60f0cc51a310efa1bd6623be.html (Accessed December 1, 2021).

[32] Cho CH Jung SW Park JY Song KS Yu KI. Is shoulder pain for three months or longer correlated with depression, anxiety, and sleep disturbance? J Shoulder Elbow Surg. (2013) 22:222-8. 10.1016/j.jse.2012.04.001 22738644

[33] Ranger TA Cicuttini FM Jensen TS Manniche C Heritier S Urquhart DM. Catastrophization, fear of movement, anxiety, and depression are associated with persistent, severe low back pain and disability. Spine J. (2020) 20:857-65. 10.1016/j.spinee.2020.02.002 32045707

[34] Wettstein M Eich W Bieber C Tesarz J. Pain intensity, disability, and quality of life in patients with chronic low back pain. does age matter? Pain Med. (2019) 20:464-75. 10.1093/pm/pny062 29701812 PMC6387985

[35] Tsuji T Matsudaira K Sato H Vietri J. The impact of depression among chronic low back pain patients in Japan. BMC Musculoskelet Disord. (2016) 17:447. 10.1186/s12891-016-1304-4 27784335 PMC5081964

[36] Kaczynski K Gambhir R Caruso A Lebel A. Depression as a mediator of the relation between family functioning and functional disability in youth with chronic headaches. Headache. (2016) 56:491-500. 10.1111/head.12709 26518249

[37] Jackson T Wang Y Wang Y Fan H. Self-efficacy and chronic pain outcomes: a meta-analytic review. J Pain. (2014) 15:800-14. 10.1016/j.jpain.2014.05.002 24878675

[38] Cheng ST Leung CMC Chan KL Chen PP Chow YF Chung JWY. The relationship of self-efficacy to catastrophizing and depressive symptoms in community-dwelling older adults with chronic pain: a moderated mediation model. PLoS ONE. (2018) 13:e0203964. 10.1371/journal.pone.0203964 30226892 PMC6143242

[39] Akbari F Dehghani M Khatibi A Vervoort T. Incorporating family function into chronic pain disability: the role of catastrophizing. Pain Res Manag. (2016) 2016:6838596. 10.1155/2016/6838596 27445620 PMC4904613

[40] Sucher J Quenstedt SR Parnes MF Brown AD. Pain centrality mediates pain self-efficacy and symptom severity among individuals reporting chronic pain. J Clin Psychol. (2020) 76:2222-31. 10.1002/jclp.23012 32567702

