# Supplementary material for: Prevalence and Associated Factors of Depressive Symptoms Among Patients With Chronic Low Back Pain: A Cross-Sectional Study
Source: Front Psychiatry. 2022 Jan 13;12:820782. doi: 10.3389/fpsyt.2021.820782 (PMC8793741; doi:10.3389/fpsyt.2021.820782)
Supplement: Supplementary file 1 [file Table_1.DOCX]

**Table S1.** Multilevel logistic regression examining individual characteristics, pain-related factors and family functioning associated with depressive symptoms

| Variables | Odds Ratio | | |
| --- | --- | --- | --- |
|  | Model 1 | Model 2 | Model 3 |
| **1. individual characteristics** |  |  |  |
| Age | 1.02^**^ | 1.01 | 1.01 |
| Sex | 1.17 | 1.12 | 0.99 |
| Marital status | 0.58^**^ | 0.68 | 0.77 |
| Educational level |  |  |  |
| Secondary school | 1.54^*^ | 1.22 | 1.25 |
| Senior high school | 1.16 | 1.02 | 0.91 |
| Junior college | 0.86 | 0.87 | 0.80 |
| University or above | 0.91 | 1.02 | 1.16 |
| Work status |  |  |  |
| Manual worker | 1.16 | 1.55 | 1.34 |
| Non-manual worker | 1.96^**^ | 1.54 | 1.24 |
| Monthly personal income | 0.51^***^ | 0.63^*^ | 0.67 |
| Medical insurance | 3.12^***^ | 1.74^*^ | 1.46 |
| 2. pain-related factors |  |  |  |
| Pain severity |  | 1.14^***^ | 1.13^***^ |
| Duration of pain |  |  |  |
| 6 months to 1 years |  | 1.41 | 1.10 |
| 1 to 3 years |  | 3.87^***^ | 2.91^***^ |
| 3 to 5 years |  | 4.67^***^ | 3.09^**^ |
| ＞5 years |  | 1.82 | 1.33 |
| Pain self-efficacy |  | 0.94^***^ | 0.94^***^ |
| 3. family function |  |  |  |
| Good family function |  |  | 0.25^***^ |
| Moderate family dysfunction |  |  | 0.47^**^ |
| **-2 log likelihood** | 1228.43 | 865.60 | 834.55 |
| **Akaike’s information criterion (AIC)** | 1252.43 | 901.60 | 874.55 |
| **Schwarz criterion (SC)** | 1313.22 | 992.80 | 975.88 |

^*^*P* value < 0.05, ^**^ *P* value < 0.01, ^***^ *P* value < 0.001.
